# Implementing the cellular mechanisms of synaptic transmission in a neural mass model of the thalamo-cortical circuitry

**DOI:** 10.3389/fncom.2013.00081

**Published:** 2013-07-04

**Authors:** Basabdatta S. Bhattacharya

**Affiliations:** Engineering Hub, School of Engineering, University of LincolnLincoln, UK

**Keywords:** neural mass model, thalamocortical circuitry, kinetic framework, brain oscillations, AMPA, GABA

## Abstract

A novel direction to existing neural mass modeling technique is proposed where the commonly used “alpha function” for representing synaptic transmission is replaced by a kinetic framework of neurotransmitter and receptor dynamics. The aim is to underpin neuro-transmission dynamics associated with abnormal brain rhythms commonly observed in neurological and psychiatric disorders. An existing thalamocortical neural mass model is modified by using the kinetic framework for modeling synaptic transmission mediated by glutamatergic and GABA (gamma-aminobutyric-acid)-ergic receptors. The model output is compared qualitatively with existing literature on *in vitro* experimental studies of ferret thalamic slices, as well as on single-neuron-level model based studies of neuro-receptor and transmitter dynamics in the thalamocortical tissue. The results are consistent with these studies: the activation of ligand-gated GABA receptors is essential for generation of spindle waves in the model, while blocking this pathway leads to low-frequency synchronized oscillations such as observed in slow-wave sleep; the frequency of spindle oscillations increase with increased levels of post-synaptic membrane conductance for AMPA (alpha-amino-3-hydroxy-5-methyl-4-isoxazolepropionic-acid) receptors, and blocking this pathway effects a quiescent model output. In terms of computational efficiency, the simulation time is improved by a factor of 10 compared to a similar neural mass model based on alpha functions. This implies a dramatic improvement in computational resources for large-scale network simulation using this model. Thus, the model provides a platform for correlating high-level brain oscillatory activity with low-level synaptic attributes, and makes a significant contribution toward advancements in current neural mass modeling paradigm as a potential computational tool to better the understanding of brain oscillations in sickness and in health.

## 1. Introduction

Neural mass computational models mimicking synchronous behavior in populations of thalamocortical neurons are often used to study brain oscillations (David and Friston, 2003; Suffczyński et al., 2004; Breakspear et al., 2006; Sotero et al., 2007; Deco et al., 2008; Izhikevich and Edelman, 2008; Pons et al., 2010; Robinson et al., 2011; de Haan et al., 2012). The term “neural mass” was coined by Freeman (1975), while the neural mass modeling paradigm is based on the mathematical framework proposed by Wilson and Cowan (1973); each cell population in a neural mass model represents a neuronal “ensemble” of mesoscopic-scale (10^4^–10^7^), which are densely packed in space and work at the same temporal-scale, so that for all practical purposes, they can be mathematically treated as a single entity (Liljenström, 2012), whence “mass”. In a seminal work, da Silva et al. (1974) used a neural mass model of a simple thalamocortical circuitry to simulate EEG (Electroencephalography) alpha rhythms (8–13 Hz). Subsequently, this model has been the basis of several research (Zetterberg et al., 1978; Stam et al., 1999; Suffczyński, 2000; Bhattacharya et al., 2011a), albeit with modifications and enhancements; of special mention is the modification introduced by Jansen and Rit (1995) where the model is expressed as a set of ordinary differential equations (ODE). This modification, in turn, has been the basis of many significant research (Wendling et al., 2002; Grimbert and Faugeras, 2006; Ursino et al., 2010). However, the computational basis of the models remain the same—the conversion from firing rate to membrane potential by excitatory and inhibitory neurotransmitters is simulated by convolution of the input from a pre-synaptic neuronal mass with an exponential function, commonly known as the “alpha function”, proposed by Rall (1967). Although the alpha function is a fair estimate of the synaptic process (Bernard et al., 1994), it does not allow an insight into the underlying cellular mechanisms of synaptic transmission associated with abnormal brain oscillations—an aspect emphasized to be crucial as an aid to research in brain disorders (McCormick, 1992; Basar and Guntekin, 2008). The importance of understanding the neuro-transmission mechanisms in slow wave synchronized as well as spindle oscillations is also discussed in several relevant experimental studies (Steriade et al., 1993; von Krosigk et al., 1993). Moreover, correlating synaptic kinetics with brain oscillatory activity has the potential to aid neuropharmacological advances in treating the diseased brain (Aradi and Erdi, 2006). Along these lines, Destexhe et al. (1998) argue that the alpha function is inappropriate for representing post synaptic events other than the originally proposed post-synaptic potential in spiking neural networks; they propose a kinetic framework as a more biologically plausible method of modeling synaptic transmission compared to the alpha function (Destexhe et al., 2002). The ability of such a modeling framework to capture the physiological properties of synaptic transmission was demonstrated by fitting the model outputs to experimental data from hippocampal slices. Moreover, kinetic modeling is reported to be computationally efficient (Destexhe et al., 1994), a vital prerequisite in large-scale computational models. Subsequently, the kinetic models of neurotransmission was used in several single-neuronal-level model-based studies—to investigate thalamic oscillations (Destexhe et al., 1996) and corticothalamic influence on brain oscillatory activity (Destexhe, 2008); to investigate network synchrony (Breakspear et al., 2003); to simulate synchronous behavior observed during *in vitro* experimental studies on ferret thalamic slice by Wang and Rinzel (1992), Golomb et al. (1994, 1996) and Wang et al. (1995).

A significant modification to current neural mass modeling framework was proposed by Suffczyński et al. (2004) by applying single-neuronal-level model based techniques. Toward this, they proposed an “ensemble” representation of the membrane conductance and post-synaptic current in a neuronal mass model of the thalamocortical circuitry; an integrator is used to generate the “ensemble” post-synaptic membrane potential. In the work presented here, a similar approach is adopted to implement the kinetic framework of synaptic transmission in neural mass models—each post-synaptic attribute is assumed to be an “ensemble” representation corresponding to a “neuronal mass”. For brevity, only two-state (“open” and “closed”) ion-channels (Destexhe et al., 1998) are considered, the desensitized state is ignored. While two-state models are a significant simplification of the very complex nature of ion channel dynamics in biology, they have shown a remarkable fit to biological data compared to more-than-two-state models (Destexhe et al., 1998, 2002). This work aims to interface an abstraction of the ion channel dynamics, such as the two-state ion channel kinetic models, with an abstraction of the population level neuronal behavior, such as neural mass models. The goal is to enable the correlation of higher-level brain dynamics observed in EEG with cellular-level dynamics.

The work is presented thus: first, the kinetic framework for modeling AMPA (α-amino-3-hydroxy-5-methyl-4-isoxazolepropionic-acid) and GABA (γ-amino-butyric-acid) receptor mediated synapses is introduced in an existing thalamocortical neural mass model (section 2); second, a qualitative comparison of the model behavior with experimental studies on ferret thalamocortical tissue reported in von Krosigk et al. (1993) as well as to single-neuronal-level model based observations reported in Golomb et al. (1996; section 3) is presented; the lack of a quantitative study is mainly to avoid erratic conclusions as difference in model structure and simulation techniques are bound to induce mismatch in numerical results. The model behavior is observed to be consistent with these studies (von Krosigk et al., 1993; Golomb et al., 1996)—The post synaptic membrane conductance in both the thalamocortical relay (TCR) and thalamic reticular nucleus (TRN) cell population plays a role in effecting a bifurcation in model behavior from spindling mode [oscillations with the characteristic waxing-and-waning pattern seen in early stages of sleep (Steriade et al., 1993; Hughes et al., 2004) as well as in alpha rhythmic oscillations during resting brain state (da Silva et al., 1973)] to a limit-cycle mode (synchronized oscillations as seen in later stages of sleep or during absence seizures). The post-synaptic membrane conductance for both AMPA and GABA in the TRN cell population is responsible for sustaining and modulating spindle oscillations in the model output. Blocking the GABA-ergic synapses in the self-inhibitory loop of the TRN cell population effects a low-frequency synchronized oscillation in the model; this is aided by the secondary-messenger-gated GABA synapses in the TCR cell population. In addition, the reverse rate of transmitter binding plays a role in increasing or decreasing the frequency of synchronized oscillations, besides functioning as a bifurcation parameter, an observation that has not been reported in experimental studies. A comparison of the simulation time of the model with previous research using neural mass models based on alpha functions show a factor of 10 improvement in simulation time. This is a dramatic improvement on computational efficiency and emphasizes the appropriateness of the model proposed herein toward building large-scale software models for investigating neuronal disorders. The observations from this study as well as issues related to the modeling approach are discussed in section 4.

## 2. Materials and methods

### 2.1. From alpha function to kinetic model: a brief outline

**Figure 1:**
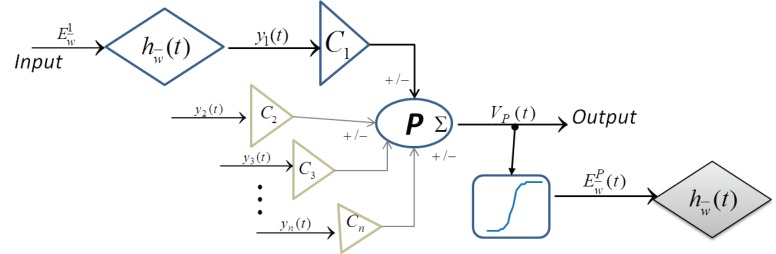
**Block diagram of a single “neuronal mass” in current state-of-the-art neural mass models**.

A single neuronal mass structure as used commonly in neural mass models is shown in Figure 1 and is defined in Equations (1–5):

(1) $$h_{\bar{w}}(t)=\frac{H_{\bar{w}}}{\tau_{\bar{w}}}\text{tex}p(-t/\tau_{\bar{w}})$$

(2) $$y_{N}(t)=\sum h_{\bar{w}}(t)⊗E_{\bar{w}}^{N}(t)$$

(3) $${\ddot{y}}_{N}(t)=\frac{H_{\bar{w}}}{\tau_{\bar{w}}}E_{\bar{w}}^{N}(t)-\frac{2}{\tau_{\bar{w}}}{\dot{y}}_{N}(t)-\frac{1}{\tau_{\bar{w}}^{2}}y_{N}(t)$$

(4) $$V_{P}(t)=\sum_{N\in \{1,\;2,\;3,\;\ldots \;n\}}C_{N}.y_{N}(t)$$

(5) $$E_{\bar{w}}^{P}(t)=S(V_{P})=\frac{2e_{0}}{1+e^{\nu (s_{0}\;-\;V_{P})}}$$

where $\bar{w}\in \{e,\;i\}$ represents pre-synaptic neuronal populations which make excitatory *(e)* and inhibitory *(i)* synapses on a post-synaptic neuronal population; $\tau_{\bar{w}}$ is the time constant and $H_{\bar{w}}$ is the amplitude of the synapse; $E_{\bar{w}}^{N}(t)$, *N* ∈ {1, 2 … *n*} is the firing frequency of an extrinsic or intrinsic cell population that is pre-synaptic to the population **P**; *C*_*N*_ is a percentage of the total number of synapses from all afferents to **P**; *V*_*P*_ is the “ensemble post-synaptic membrane potential”; $E_{\bar{w}}^{P}$ is the “ensemble firing rate” of **P** and is defined by a sigmoid function where 2*e*_0_ is the maximum firing rate of the population, *s*_0_ is the threshold potential at which the neurons spike and ν is the sigmoid steepness parameter.

#### 2.1.1. A modified neural mass representation

**Figure 2:**
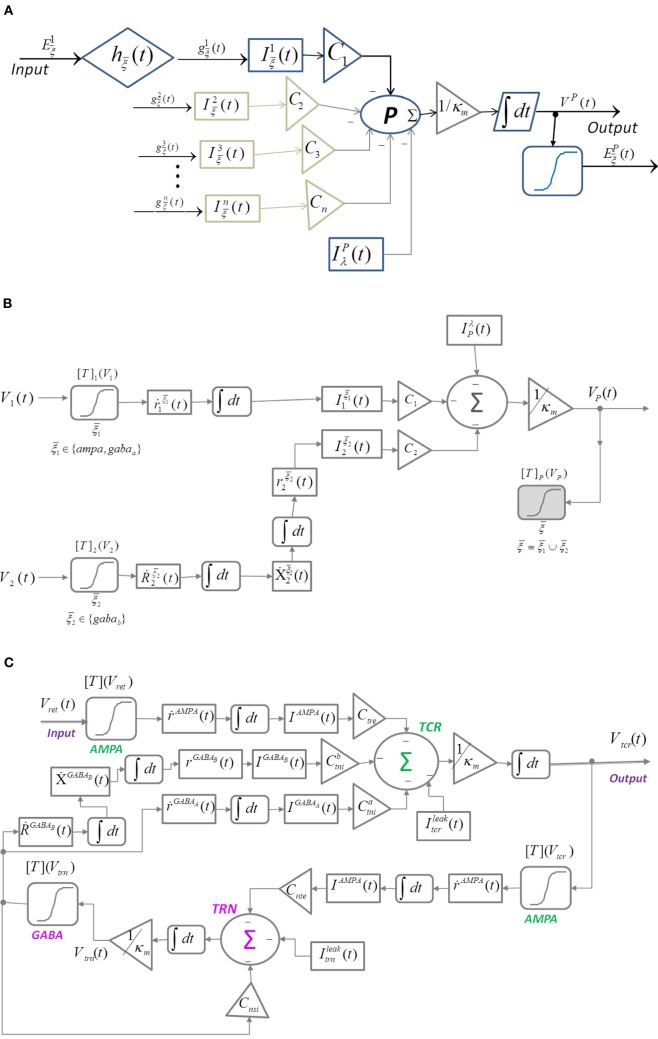
**Block diagram of **(A)** Suffczyński et al. (2004)'s modification of the structure in Figure 1 by introducing “ensemble” representation of post-synaptic membrane conductance and current. (B)** Neuronal mass structure with the kinetic framework implemented for modeling synaptic transmission as an alternative to the alpha function ($h_{\bar{w}}(t)$ in Figure 1). A diagrammatic representation of the ion-channel kinetics during synaptic transmission is presented in Figures 3A,B. **(C)** A thalamocortical circuitry implementing the modified neuronal mass representation in **(B)**.

In a recent work, Suffczyński et al. (2004) modified the neural mass representation of a cell population and introduced post-synaptic current mediated by the ligand-gated glutamatergic receptors AMPA, and the ligand- and secondary-messenger-gated GABA-ergic receptors GABA_*A*_ and GABA_*B*_, respectively. The input $E_{\bar{\xi }}^{N}(t)$, $\bar{\xi }\in \{\text{AMPA},{\text{ GABA}}_{A},{\text{ GABA}}_{B}\}$, is the firing rate of an excitatory (AMPA) or inhibitory (GABA_*A*_ and GABA_*B*_) pre-synaptic neuronal population *N* ∈ {1, 2 …, *n*}. The model (Figure 2A) is defined in Equations (6–11):

(6) $$h_{\bar{\xi }}(t)=H_{\bar{\xi }}\left(\text{exp}\left(-t/\tau_{\bar{\xi }}^{a}\right)-exp\left(-t/\tau_{\bar{\xi }}^{b}\right)\right),\;\tau_{\bar{\xi }}^{b}>\tau_{\bar{\xi }}^{a}$$

(7) $$g_{\bar{\xi }}^{N}(t)=\sum h_{\bar{\xi }}(t)⊗E_{\bar{\xi }}^{N}(t)$$

(8) $${\ddot{g}}_{\bar{\xi }}^{N}(t)=\frac{1}{\tau_{\bar{\xi }}^{a}\tau_{\bar{\xi }}^{b}}[H_{\bar{\xi }}\left(\tau_{\bar{\xi }}^{a}-\tau_{\bar{\xi }}^{b}\right)E_{\bar{\xi }}^{N}(t)-\left(\tau_{\bar{\xi }}^{a}+\tau_{\bar{\xi }}^{b}\right){\dot{g}}_{\bar{\xi }}^{N}(t)-g_{\bar{\xi }}^{N}(t)]$$

(9) $$I_{\bar{\xi }}^{N}(t)=g_{\bar{\xi }}^{N}(t)\left(V^{P}(t)-V_{\bar{\xi }}\right)$$

(10) $$\kappa_{m}{\dot{V}}^{P}(t)=-\sum_{N\in \{1,\;2,\;3,\;\ldots ,\;n\}}C_{N}.I_{\bar{\xi }}^{N}(t)-I_{\lambda }(t)$$

(11) $$I_{\lambda }(t)=g_{\lambda }\left(V^{P}(t)-V_{\lambda }\right)$$

where $h_{\bar{\xi }}(t)$ is the synaptic transmission function with $\tau_{\bar{\xi }}^{a}$ and $\tau_{\bar{\xi }}^{b}$ as the rise and decay times, respectively; $g_{\bar{\xi }}^{N}$ denote the post-synaptic “ensemble” membrane conductance; $V_{\bar{\xi }}$ is the reversal potential for the synapse mediated by $\bar{\xi }$; *V*^*P*^ is the ensemble post synaptic membrane potential of the population **P** due to PSC from all pre-synaptic cell populations *N* ∈ {1, 2 …, *n*}; κ_*m*_ is the ensemble membrane capacitance; *C*_*N*_ is the synaptic connectivity parameter; *I*_λ_, *g*_λ_ and *V*_λ_ are the ensemble leakage current, conductance and reversal potential, respectively for **P**. The ensemble firing rate $E_{\bar{\xi }}^{P}(t)$ is as defined in Equation (5) and is the pre-synaptic firing rate input to other neuronal populations.

#### 2.1.2. Introducing kinetic model of synapses in a neural mass representation

The single neuronal mass structure presented in Figures 1, 2A is modified by replacing the alpha function with kinetic models of AMPA, GABA_*A*_, and GABA_*B*_ synapses; the enhanced representation (Figure 2B) is defined in Equations (12–19):

(12) $${\left[T\right]}_{\chi }(V_{\chi })=\frac{T_{\text{max}}}{1+e^{-\frac{V_{\chi }\;-\;\theta_{s}}{\sigma_{s}}}}$$

(13) $$\frac{dr_{\chi }^{\bar{\xi_{1}}}(t)}{dt}=\alpha^{\bar{\xi_{1}}}{\left[T\right]}_{\chi }\left(1-r_{\chi }^{\bar{\xi_{1}}}(t)\right)-\beta^{\bar{\xi_{1}}}r_{\chi }^{\bar{\xi_{1}}}(t)$$

(14) $$\frac{dR_{\chi }^{\bar{\xi_{2}}}(t)}{dt}=\alpha^{\bar{\xi_{2}}}{\left[T\right]}_{\chi }\left(1-R_{\chi }^{\bar{\xi_{2}}}(t)\right)-\beta^{\bar{\xi_{2}}}R_{\chi }^{\bar{\xi_{2}}}(t)$$

(15) $$\frac{d[X](t)}{dt}=\alpha^{\bar{\xi_{2}}}R_{\chi }^{\bar{\xi_{2}}}(t)-\beta^{\bar{\xi_{2}}}[X](t)$$

(16) $$r_{\chi }^{\bar{\xi_{2}}}((t))=\frac{{\left[X\right]}^{n}(t)}{{\left[X\right]}^{n}(t)+K_{d}}$$

(17) $$I_{\chi }^{\bar{\xi }}(t)=g^{\bar{\xi }}r_{\chi }^{\bar{\xi }}(t)\left(V_{P}(t)-V^{\bar{\xi }}\right)$$

(18) $$\kappa_{m}\frac{dV_{P}(t)}{dt}=-\sum_{\chi \;\in \;\{1,\;2\}}I_{\chi }^{\bar{\xi }}(t).C_{\chi }-I_{P}^{\lambda }(t)$$

(19) $$I_{P}^{\lambda }(t)=g_{P}^{\lambda }\left(V_{P}(t)-V_{P}^{\lambda }\right)$$

**Figure 3:**
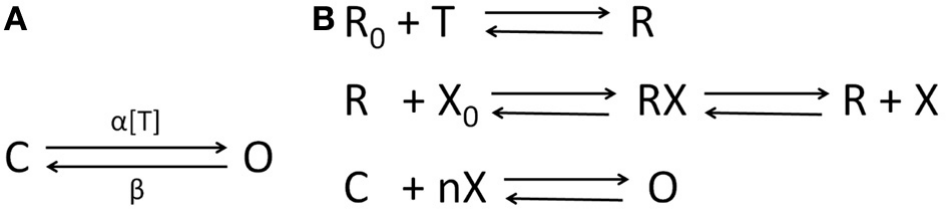
**The state transition diagrams for **(A)** AMPA and GABA_*A*_ neuro-receptor dynamics defined in Equation (13);** α **and** β **are rate of transitions between the two states and** α **is a function of the transmitter concentration in the synaptic cleft [*T*] defined in Equation (12); **(B)** GABA_*B*_ neuro-transmission as defined in Equations (14–16)—the neurotransmitter *T* binds to the inactivated receptor *R*_0_; a fraction of activated receptors *R* act as a catalyst to transform the G-protein from an inactivated form *X*_0_ to an activated form *X*, which binds at *n* independent sites to open a fraction of the ion channels.** The desensitized state of the ion-channels are ignored in this work for brevity (see Destexhe et al., 1998 for a detailed comparison of kinetic models with more than two-states).

Let *V*_χ_, χ ∈ {1, 2} be the “ensemble” membrane potential of two pre-synaptic neuronal population that are afferent to the post-synaptic population **P** such that the synapses made by χ = 1 is mediated by a ligand-gated receptor $\bar{\xi_{1}}\in \{\text{AMPA},{\text{ GABA}}_{A}\}$ while that made by χ = 2 is mediated by a secondary-messenger-gated receptor $\bar{\xi_{2}}\in \{{\text{GABA}}_{B}\}$. The concentration of neurotransmitters [*T*]_χ_ in the synaptic cleft is defined as a function of *V*_χ_ and is expressed by a sigmoid function (Equation 12) where *T*_max_ is the maximum neuronal concentration in the synaptic cleft and is well approximated by 1 mM (Destexhe et al., 1998), θ_*s*_ represents the threshold at which [*T*] = 0.5*T*_max_ and σ_*s*_ denote the steepness of the sigmoid. The proportion of open ion-channels due to the bound receptors ${\bar{\xi }}_{1}$ on the ensemble membrane of the post-synaptic cell population corresponding to the synapse made by the population χ = 1 is defined in Equation (13) where $\alpha^{\bar{\xi_{1}}}$ and $\beta^{\bar{\xi_{1}}}$ are the forward and backward rate constants, respectively for transmitter binding. The transition diagram is shown in Figure 3A. However, GABA_*B*_ mediated synapses, unlike AMPA and GABA_*A*_ synapses, activate G-proteins which in turn act as the “secondary messengers” and initiate the opening of ion channels. The process is defined in Equations (14–16) where $R_{\chi }^{\bar{\xi_{2}}}$ is the fraction of activated $\bar{\xi_{2}}$ receptors, which acts as a catalyst in activating the secondary-messenger G-protein (guanine nucleotide–binding proteins); [X] is the concentration of the activated G-protein; $r_{\chi }^{\bar{\xi_{2}}}$ is the fraction of open ion channels caused by binding of X with independent binding sites; $\alpha^{\bar{\xi_{2}}}$ and $\beta^{\bar{\xi_{2}}}$ are the binding rate constants; *n* is the number of bound receptor sites and K_*d*_ is the dissociation constant of binding of X with the ion channels. The transition diagram of this process is shown in Figure 3B. The resulting ensemble PSC mediated by the receptor $\bar{\xi }\equiv \bar{\xi_{1}}\cup \bar{\xi_{2}}$ due to a synapse from the pre-synaptic population χ is defined in Equation (17) where $g^{\bar{\xi }}$ and $V^{\bar{\xi }}$ are the maximum conductance and reverse potential, respectively corresponding to ξ mediated synapse. *V*_*P*_ (Equation 18) is the ensemble post-synaptic potential (PSP) of **P**, where κ_*m*_ is the ensemble membrane capacitance of **P**, *C*_χ_, χ ∈ {1, 2} is the synaptic connectivity parameter. *I*^λ^_*P*_ (Equation 19) is the ensemble leak current of the post-synaptic membrane, where *g*^λ^_*P*_ and *V*^λ^_*P*_ are conductance and reverse potential, respectively, corresponding to “non-specific” leak (Golomb et al., 1996; Suffczyński et al., 2004) in the ensemble membrane of the post synaptic cell population. In the following section, we implement this framework in a neural mass model of the thalamocortical circuitry.

### 2.2. Neural mass model of a thalamocortical circuitry with kinetic synapses

The thalamocortical circuitry is shown in Figure 2C and consists of the two thalamic cell populations that communicate with the cortex viz. the TCR and TRN. The third group of cells viz. the Interneurons (IN) participate in intra-thalamic communications and are ignored here for brevity. The synaptic structure and connectivity are informed from experimental data based on the dorsal thalamic Lateral Geniculate Nucleus (LGNd) (Horn et al., 2000). The input to the model is assumed to be the ensemble membrane potential of pre-synaptic retinal cells (*V*_ret_) in a resting state with no sensory input and is simulated using a Gaussian white noise (da Silva et al., 1973). The TCR cells make AMPA receptor mediated glutamatergic synapses on the TRN cells (other types of glutamatergic receptors are ignored in this work for brevity); the TRN cells make GABA-ergic synapses on the TCR cells mediated by both the ligand-gated GABA_*A*_ and the secondary-messenger-gated GABA_*B*_ receptors. Furthermore, the TRN cells make GABA_*A*_ receptor mediated synapses within the population. The model is defined in Equations (20–27); all variables and parameters in the model are assumed to be the ensemble representation corresponding to a neural mass:

(20) $${\left[T\right]}_{\bar{\Psi }}(V_{\bar{\Psi }}(t))=\frac{T_{\text{max}}}{1+exp\left(-\frac{V_{\bar{\Psi }}(t)-\theta_{s}}{\sigma_{s}}\right)}$$

(21) $$\frac{dr_{\bar{\Psi }}^{{\bar{\eta }}_{1}}(t)}{dt}=\alpha^{{\bar{\eta }}_{1}}{\left[T\right]}_{\bar{\Psi }}\left(1-r_{\bar{\Psi }}^{{\bar{\eta }}_{1}}(t)\right)-\beta^{{\bar{\eta }}_{1}}r_{\bar{\Psi }}^{{\bar{\eta }}_{1}}(t)$$

(22) $$\frac{dR_{\bar{\Psi }}^{\bar{\eta_{2}}}(t)}{dt}=\alpha_{1}^{\bar{\eta_{2}}}{\left[T\right]}_{\bar{\Psi }}\left(1-R_{\bar{\Psi }}^{\bar{\eta_{2}}}(t)\right)-\beta_{1}^{\bar{\eta_{2}}}R_{\bar{\Psi }}^{\bar{\eta_{2}}}(t)$$

(23) $$\frac{d[X](t)}{dt}=\alpha_{2}^{\bar{\eta_{2}}}R_{\bar{\Psi }}^{\bar{\eta_{2}}}(t)-\beta_{2}^{\bar{\eta_{2}}}[X](t)$$

(24) $$r_{\bar{\Psi }}^{\bar{\eta_{2}}}((t))=\frac{{\left[X\right]}^{n}(t)}{{\left[X\right]}^{n}(t)+K_{d}},$$

(25) $$I_{\bar{\Psi }}^{\bar{\eta }}(t)=g^{\bar{\eta }}r_{\bar{\Psi }}^{\bar{\eta }}(t)\left(V_{\bar{ϒ}}(t)-V^{\bar{\eta }}\right)$$

(26) $$\kappa_{m}\frac{dV_{\bar{ϒ}}(t)}{dt}=-\sum_{\bar{\Psi }\;\in \;\{\text{ret},\text{ trn},\text{ trn}\}}I_{\bar{\Psi }}^{\bar{\eta }}(t).C_{\bar{u}\bar{v}\bar{w}}-I_{\bar{ϒ}}^{\lambda }(t),$$

(27) $$I_{\bar{ϒ}}^{\lambda }(t)=g_{\bar{ϒ}}^{\lambda }(V_{\bar{ϒ}}(t)-V_{\bar{ϒ}}^{\lambda }),$$

where $\bar{\Psi }\in \{\text{ret},\text{ trn},\text{ trn}\}$ represent the afferent cell populations; $\bar{ϒ}=\{\text{trn},\text{ trn}\}$ represent the efferent cell populations; ${\bar{\eta }}_{1}\in \{\text{AMPA},{\text{ GABA}}_{A}\}$, ${\bar{\eta }}_{2}\in \{{\text{GABA}}_{B}\}$, $\bar{\eta }\equiv {\bar{\eta }}_{1}\cup {\bar{\eta }}_{2}$; $C_{\bar{u}\bar{v}\bar{w}}$ are connectivity parameters where $\bar{u}\in \{t,\;n\}$ and $\bar{v}\in \{r,\;t,\;n,\;s\}$ denote the post-synaptic and pre-synaptic cell populations, respectively of the retina (*r*), TCR (*t*), TRN (*n*), while *s* denote an intra-population afferent; $\bar{w}\in \{e,\;i\}$ represent an excitatory (*e*) or an inhibitory (*i*) synapse. All other parameter nomenclatures are as defined in section 2.1. The initial parameter values are mentioned in Table 1.

**Table 1:** **Initial values of the parameters defined in Equations (21–27)**.

**Neuroreceptors** →		**AMPA**	**GABA_*A*_**	**GABA_*B*_**
**Units**↓				
**(A) NEUROTRANSMISSION PARAMETERS**				
mM.msec^−1^		$\alpha^{{\bar{\eta }}_{1}}=2$	$\alpha^{{\bar{\eta }}_{1}}=2$	$\alpha_{1}^{{\bar{\eta }}_{2}}=0.02$
				$\alpha_{2}^{{\bar{\eta }}_{2}}=0.03$
msec^−1^		$\beta^{{\bar{\eta }}_{1}}=0.1$	$\beta^{{\bar{\eta }}_{1}}=0.08$	$\beta_{1}^{{\bar{\eta }}_{2}}=0.05$
				$\beta_{2}^{{\bar{\eta }}_{2}}=0.01$
mS		$g^{\bar{\eta }}=0.1$	$g_{\text{TRN to TCR}}^{\bar{\eta }}=0.1$	$g^{\bar{\eta }}=0.06$
			$g_{\text{TRN to TRN}}^{\bar{\eta }}=0.2$		
mV		$V^{\bar{\eta }}=0$	$V_{\text{TRN to TCR}}^{\bar{\eta }}=-85$	$V^{\bar{\eta }}=-100$
			$V_{\text{TRN to TRN}}^{\bar{\eta }}=-75$		
				*K*_*d*_ = 100
				*n* = 4
**(B) CELL MEMBRANE PARAMETERS**				
	**TCR**		**TRN**	
${\text{g}}_{\bar{ϒ}}^{\lambda }\;(\text{mS})$	0.01		0.01	
${\text{V}}_{\bar{ϒ}}^{\lambda }\;(\text{mV})$	−55		−72.5	
*V*_rest_ (mV)	−61		−84	
**(C) CONNECTIVITY PARAMETERS**				
**Efferents** →	**TCR**	**TRN**		**Retinal**
**Afferents** ↓		**GABA_*A*_**	**GABA_*B*_**	
TCR	X	C^*a*^_tni_	C^*b*^_tni_	C_tre_
		$\frac{3}{4}\text{ of }30.9$	$\frac{1}{4}\text{ of }30.9$	7.1
TRN	C_nte_	C_nsi_	X	X
	35	20		

*Data in (A) and (B) are as in Golomb et al. (1996) and Suffczyński et al. (2004). In Equation (20), both θ_s_ and σ_s_ act as bifurcation parameters in the model (see Bhattacharya et al., 2012). However, the emphasis here is on post-synaptic membrane attributes as in von Krosigk et al. (1993) and Golomb et al. (1996). Thus these parameters (θ_s_ =-35 and σ_s_ = 2) are set by trial and error at values just before the model undergoes bifurcation from a “point-attractor” mode to a “limit-cycle” mode, based on a recent study where we observed rich model dynamics and power spectral behavior around the bifurcation point (Bhattacharya et al., 2013); T_max_ = 1 mM (Destexhe et al., 1994). The input noise mean μ = −45 mV and standard deviation φ = 20 mV^2^ are set by trial and error and represents the resting state membrane potential fluctuations in retinal cells. While the total number of GABA-ergic synaptic count on TCR cells is reported as 30.9%, specific data on GABA_*A*_ and GABA_*B*_ are not available in literature to the best of our knowledge. Thus, values for C^a^_tni_, C^b^_tni_ and C_nsi_ in (C) are selected, within the reported biological range [see Bhattacharya et al. (2011b) for details], when the model output showed an increased frequency content within the theta (4–7 Hz) and alpha (8–13 Hz) bands. C_tre_ and C_nte_ are as in Bhattacharya et al. (2011b). All variables in the ODEs are initialized to an arbitrarily small value 0.0002*.

## 3. Results

The ODEs are solved using the 4th/5th order Runge-Kutta-Fehlberg method (RKF45) in Matlab for a total duration of 600 s (10 min) at a resolution of 1 ms. The output voltage time series is averaged over 20 simulations, each simulation run with different seed for the noisy input. For frequency analysis, an epoch from 100–599 s of the output signal is sampled every 4 ms (250 Hz) and bandpass filtered between 3.5–14 Hz with a Butterworth filter of order 10. Short Time Fourier Transform (STFT) is done with a Hamming window of duration 10 s and overlap of 50%.

**Figure 4:**
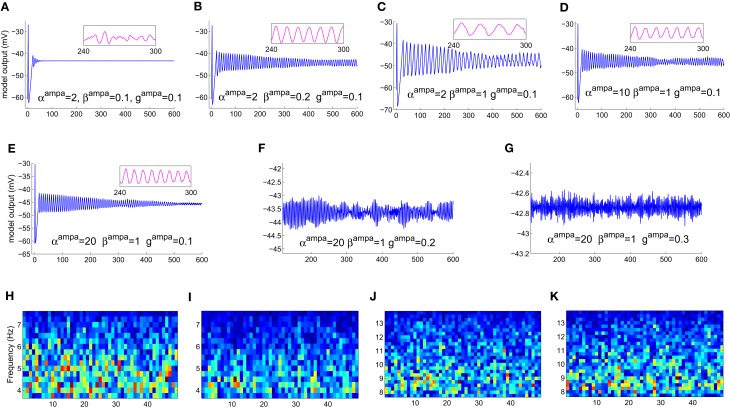
**The model output time series with **(A)** all parameters at their initial values as in Table 1. (B)** The model displays a bifurcation in output behavior when β^ampa^ is increased to 0.2; **(C)** a further increase in the parameter shows sustained oscillations with increased magnitude and decreased frequency. The frequency behavior may be observed qualitatively in the embedded line plots displaying the respective time-series in each plot for an arbitrarily selected period of 60 s. **(D)** An increase in α^ampa^ has a reverse effect resulting in reduced magnitude and increased frequency in the limit-cycle mode; **(E)** further increase in the parameter do not show any significant effect on the magnitude or the limit-cycle behavior while there is a slight increase in frequency. **(F)** Maintaining these modified values of α^ampa^ = 20 and β^ampa^ = 1, an increase in *g*^ampa^ brings a bifurcation in model behavior from a limit-cycle mode to a “spindling” mode. A “zoomed-in” plot from the 3rd minute to the 9th minute is shown; the initial transient oscillations are neglected. **(G)** The frequency of spindle oscillations increase with increasing values of *g*^ampa^. This is indicated by a distinct **(H,I)** decrease (more blue pixels) in theta band components and **(J, K)** increase (more red, orange and yellow pixels) in alpha band components in the corresponding output time series plots. The abscissa in the figures denote (**A–G**) time (seconds) (**H–K**) time windows (seconds).

The model displays a point-attractor mode behavior (initial transient oscillations before settling down to a low amplitude noisy output, which reflects the noisy input of the model) corresponding to initial parameter values (Figure 4A). There is a behavioral transition in the model to a limit cycle mode with increasing values of β^ampa^, which correlates with a decrease in the fraction of open ion channels in the post-synaptic ensemble membrane (Figures 4B,C). Varying α^ampa^, on the other hand, does not affect the model behaviour (Figures 4D,E). A transition from the limit cycle mode to a spindling mode is effected in the model by increasing *g*^ampa^, the post-synaptic membrane conductance for AMPA mediated synapses in both TCR and TRN cell population, and shown in Figures 4F,G. STFT of the output time series indicates the non-stationary behavior of the model (Figures 4H–K). A decrease and increase, respectively of the theta and alpha band components imply an overall increase in frequency with increasing values of *g*^ampa^ ≡ {*g*^ampa^_TCR_, *g*^ampa^_TRN_}, where *g*^ampa^_TCR_ and *g*^ampa^_TRN_ correspond to the incoming signal from the retina (to the TCR) and TCR (to the TRN), respectively in the model. These observations are consistent with similar reports of a transition in the state of the model output with increasing values of *g*^ampa^ in Golomb et al. (1996; pp. 756–757), accompanied by an abrupt increase in the ratio of the frequency of oscillation of the TCR and the TRN cell populations; we have not studied the latter aspect in this work. A more detailed study on the model presented herein where *g*^ampa^_TCR_ and *g*^ampa^_TRN_ are varied separately specify the *g*^ampa^_TCR_ as the control parameter that causes a bifurcation in the model output from a limit cycle mode to the spindling mode with an increase in its value. On the other hand, the *g*^ampa^_TRN_ does not effect any behavioral change in the model output, rather, it is effective in increasing the inter-spindle frequency with an increase in its value when the model is in a spindling mode. This observation implies that a change in AMPA receptor related attributes in the TRN plays a role in modulating thalamocortical spindle oscillations, which finds strong support in the experimental study by von Krosigk et al. (1993), where “activation of AMPA-kainate receptors on the PGN” (Peri-geniculate nucleus—the part of the TRN associated with the LGNd) is described as “critical to the generation of spindle waves”. Furthermore, this observation is in line with the TRN being widely implicated as being the key “ingredient” in the generation of thalamocortical spindle oscillations (McCormick, 1992; Steriade et al., 1993).

**Figure 5:**
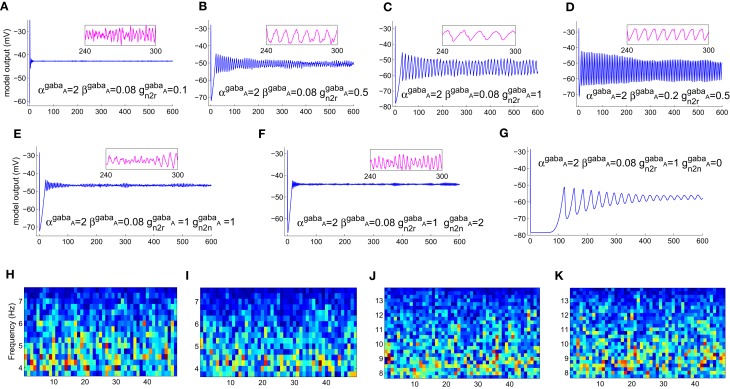
**(A)** The model output (corresponding to the “zoomed-in” plot in Figure 4G) when α^ampa^ = 20, β^ampa^ = 1, *g*^ampa^ = 0.3). Retaining these parameter values, **(B)** increasing *g*^gaba_*A*_^_*n2r*_, where “*n2r*” denotes the TRN to TCR pathway, from its initial value effects a bifurcation in model behavior from a spindling mode to a limit-cycle mode, indicating highly synchronized oscillations in the thalamocortical circuitry. **(C)** Synchronization increases with increase in *g*^gaba_*A*_^_*n2r*_, indicated by increased magnitude and decreased frequency of oscillation. For *g*^gaba_*A*_^_*n2r*_ ≥slant 0.5 [approximate point of bifurcation, shown in **(B)**], **(D)** increasing β^gaba_*A*_^ effects an increase in frequency within the limit-cycle mode, while **(E)** increasing *g*^gaba_*A*_^_*n2n*_, where “*n2n*” denotes the self inhibitory pathway of the TRN, effects a transition from the limit-cycle mode to the spindling mode. **(F)** Further increase in *g*^gaba_*A*_^_*n2n*_ causes an increase in the frequency of the spindle oscillations, indicated by **(H,I)** a decrease (more blue pixels) in theta band components and **(J,K)** increase (more red, orange and yellow pixels) in alpha band components in the corresponding output time series plots. **(G)** Blocking of *g*^gaba_*A*_^_*n2n*_ shows a very low-frequency (≈0.03 Hz) synchronized oscillation whose magnitude decreases with time. The abscissa in the figures denote (**A–G**) time (seconds) (**H–K**) time windows (seconds).

Varying the GABA-ergic synaptic attributes when the model is in a point-attractor mode does not show any change in model behavior. When the model is in a spindling mode (Figure 5A), increased synchronization within the limit cycle mode with increasing values of *g*^gaba_*A*_^_TRN to TCR_ (Figures 5B,C) is observed. An increase in the parameter β^gaba_*A*_^ affects the output only when the model is in a limit-cycle mode and counters the effect of increase in *g*^gaba_*A*_^_TRN to TCR_ (Figure 5D). However, varying α^gaba_*A*_^ does not affect the model output. For *g*^gaba_*A*_^_TRN to TCR_ ≥slant 0.5, which is the approximate bifurcation point (Figure 5B), increasing *g*^gaba_*A*_^_TRN to TRN_ causes the model to revert back to the spindling mode; the frequency of the inter-spindle oscillations increase with increasing values of the parameter (Figures 5E,F). This is also indicated by a decrease (Figures 5H,I) and increase (Figures 5J,K) of theta and alpha band components respectively in the STFT of the output time series. In other words, decreasing values of the parameter *g*^gaba_*A*_^_TRN to TRN_ causes increased synchronization within the spindling mode behavior of the model along with a decrease in the inter-spindle frequency. However, blocking *g*^gaba_*A*_^_TRN to TRN_ effects a switch in the model behavior to a very low-frequency oscillatory state (Figure 5G). These results are consistent with experimental findings (von Krosigk et al., 1993) where application of GABA_*A*_ inhibitor either “abolished spindle waves or decreased within-spindle frequency,” which correspond to the condition of either blocking or decreasing, respectively of *g*^gaba_*A*_^_TRN to TRN_ in our model. Thus, the model implicate the intra-TRN synaptic activity to be a key factor in sustaining spindle oscillations in the thalamocortical circuitry, an observation which conforms to those made in Golomb et al. (1996; p. 755). Furthermore, a “frequency jump” with increasing *g*^gaba_*A*_^, and associated transition in model behavior is also reported in Golomb et al. (1996; see Figure 7) as a comparative study between the TCR and TRN cells. This is similar with the increase in frequency of the spindle oscillations corresponding to increasing *g*^gaba_*A*_^_TRN to TRN_ in the present model, although we have not done a comparative study with the TRN cell population behavior. However, the current study implicate the increased post-synaptic conductance for GABA_*A*_ receptors in the TCR cell population (*g*^gaba_*A*_^_TRN to TCR_) to play a significant role in effecting state-transition between spindle and slow-wave oscillations, an observation that is yet to find support from experimental or model-based studies.

A quiescent state is observed corresponding to blocking either AMPA (*g*^ampa^ = 0) or both GABA_*A*_ (*g*^gaba_*A*_^_TRN to TCR_ = 0) and GABA_*B*_ (*g*^gaba_*B*_^_TRN to TCR_ = 0) mediated synapses in the TRN to TCR pathway (not shown). This is consistent with both experimental (von Krosigk et al., 1993) and model-based (Golomb et al., 1996) studies. The role of the synaptic parameters in the GABA_*B*_ pathway in our model was minimal—to sustain a non-quiescent model behavior with blockage of GABA_*A*_; to sustain a high amplitude of limit-cycle oscillations in the model. Again, this is in agreement with experimental studies (von Krosigk et al., 1993), where activation of GABA_*B*_ receptors are reported as “not essential” for generating synchronized oscillations, while application of GABA_*B*_ antagonist abolished “evoked or spontaneous slowed oscillations.” The model in Golomb et al. (1996; see in Discussion p. 763) is also mentioned as being consistent with these experimental results.

In a recent work (Bhattacharya et al., 2012), a simple neural mass model implementing kinetic modeling for synaptic transmission is presented; the synaptic connectivity parameters in the model correlate directly to that of an alpha function based neural mass model [modified Alpha Rhythm model (modARm) from Bhattacharya et al. (2011a)]. The model behavior is studied corresponding to changes in the synaptic connectivity parameters as well as transmitter concentration related parameters, and a relevant comparison is made with the modARm. However, the model presented in this work has a larger set of synaptic connectivity parameters; model behavior corresponding to this parameter space and its usefulness in understanding neurological disorders will be the topic of a future work.

## 4. Discussion

The work presented here explores a novel approach toward correlating current neural mass model based studies with underlying cellular mechanisms during synaptic transmission. The aim is to underpin the synaptic correlates of abnormal brain oscillations in neurological and psychiatric disorders such as observed in Electroencephalogram (EEG). A kinetic framework for modeling AMPA and GABA receptor mediated synapses is implemented in an existing thalamocortical neural mass model consisting of an excitatory and an inhibitory neural mass, representing cell populations of the thalamocortical relay (TCR) and the thalamic reticular nucleus (TRN), respectively. Parameters in the model are assumed to be “ensemble” representations of the corresponding attributes in a single neuron. A preliminary observation is made on the model behavior by varying the parameters corresponding to the post-synaptic membrane conductance of the cell populations as well as the forward and reverse rates of synaptic reaction; of specific interest is the transition of the model behavior between the spindle oscillatory mode and the limit-cycle mode, the latter resembling the slow-wave (high-amplitude, low-frequency) synchronized oscillations that are signatures of absence seizures as well as slow-wave sleep. Furthermore, only the alpha (8–13 Hz) and theta (4–7 Hz) frequency bands of the output power spectra are studied here, as EEG alpha and theta bands are believed to have a strong correlation with thalamocortical oscillations (Hughes et al., 2004).

The results indicate that: (1) The post synaptic membrane conductance for both AMPA and GABA_*A*_ receptors in the TRN cell population play a role in sustaining spindle oscillations of the TCR cell population (the model output). (2) Blocking the GABA_*A*_ mediated synapses in the self-inhibitory feedback pathway of the TRN cell population effects synchronized oscillations with high amplitude and increased time-period of oscillation (≈0.03 Hz). (3) The post-synaptic membrane conductance for GABA_*B*_ in the TCR cell population does not play any role in generating or sustaining spindle oscillations, but is responsible for sustaining the slow-wave oscillations in the model associated with blocking of the intra-TRN GABA_*A*_ synapses. (4) Blocking both GABA_*A*_ and GABA_*B*_ or only the AMPA mediated synapses in the TCR cell population results in a quiescent model output. These findings are consistent with *in vitro* studies based on multiple unit recordings from ferret thalamic slices (von Krosigk et al., 1993) as well as single-neuron-level model based studies (Golomb et al., 1996). In addition, this study identifies—(a) the reverse rate of transmitter binding as an important attribute in effecting thalamocortical synchronized oscillations that can be induced in the model by increasing (decreasing) the fraction of open channels due to GABA_*A*_ (AMPA) mediated synapses in the TCR (TRN) cell populations; (b) the post-synaptic membrane conductance for GABA_*A*_ in the TCR cell population as a control parameter for effecting a behavioral transition in the model.

It may be noted that the above-mentioned observations are only a qualitative comparison with single-neuron-level model-based (Golomb et al., 1996) and experimental (von Krosigk et al., 1993) studies; a drawback of the current work is a lack of quantitative comparison with these studies. The neural mass model presented in this work is at a mesoscopic scale, representation of a population of ≈10^4^−10^7^ neurons, unlike that in Golomb et al. (1996), which is at single-neuronal-level. Similarly, the multiple unit recording based study in von Krosigk et al. (1993) observes neuronal behavior of either a single neuron or a population of <10^2^ neurons. In addition, the modeling and simulation methods in the current work and that in Golomb et al. (1996) are not similar. Thus, a quantitative comparison of the current work with these studies may lead to erroneous conclusions. However, model validation with experimental data is a crucial criteria when investigating brain disorders. Along these lines, an ongoing work is investigating ways to validate the model presented herein with EEG data, and will be the topic of a future study.

The model structure in the current work is a considerably simplified representation of the thalamocortical circuitry. The role of the thalamocortical circuitry in generating slow wave brain oscillations is discussed at length in Steriade et al. (1993), based on *in vivo* and *in vitro* studies. More recently, three parameters in the thalamo-cortico-thalamic loop viz. the cortico-thalamic, thalamo-cortical and intra-thalamic pathways are specified in Breakspear et al. (2006) for generating instabilities in the thalamocortical circuitry, leading to synchronized oscillations such as seen during absence seizures. Furthermore, a non-linear dynamical analysis of the model is shown to predict seizure onset by validating with patient EEG data. In a previous research (Bhattacharya et al., 2011b), we have proposed a more elaborate alpha-function based neural mass model that have considered these vital pathways in the thalamocorticothalamic loop. Also, we have performed a non-linear dynamical analysis of a simple thalamocortical model based on alpha functions in Bhattacharya et al. (2013) to understand EEG power spectra abnormalities associated with several neurological disorders. Such research directions will be considered as an extended work based on the model presented herein.

It is worth mentioning here that biologically plausible parameterizations has been a major constraint in neural mass modeling of brain dynamics. This is largely due to insufficient experimental data, published or otherwise, as well as to a lack of “homogeneity” of published data from different experimental laboratories. The trend thus far has been to use biologically plausible data if and when available; otherwise, i.e., for parameter values that cannot be availed from experimental data, the models are tuned to estimated parameter values which provide a desirable output in context to the objectives of the research [the reader may refer to Robinson et al. (2004) for a model parameterizations related work and discussion]. Thus, the model in Breakspear et al. (2006) was based on neurophysiological parameters obtained from Robinson et al. (2002), which in turn are based on inverse parameterizations during model validation with EEG data from patients of epileptic seizures. The parameterizations of the model presented in this work is largely based on neurophysiological parameters obtained from experimental studies: the cellular-level parameters, including those of the synaptic kinetics, are based on *in vitro* studies and model-based studies of thalamocortical tissue by von Krosigk et al. (1993) and Golomb et al. (1996), respectively; the model connectivity parameters are based on experimental studies of the cat and rat thalamus obtained from Horn et al. (2000); Sherman and Guillery (2001). On the other hand, the extrinsic (retinal) input and neuro-transmitter concentration parameter values are adjusted to maintain a “dynamically active” model behavior (this is as opposed to a continuous “quiescent” state of the model corresponding to certain parameter values, and does not conform to biology). However, technological advances in the field of neuro-imaging during the last decade such as functional Magnetic Resonance Imaging (fMRI), Diffusion Tensor Imaging (DTI) and Transcanial Magnetic Stimulation (TMS) are paving the way for biologically-realistic mapping of parameter values in computational models; for example as in Izhikevich and Edelman (2008).

The observations made herein support the motivation toward this preliminary work, which is to correlate higher-level brain dynamics with underlying cellular-level synaptic mechanisms. It may be noted that in all our previous works using alpha function based neural mass models, the emphasis has been on studying the model behavior with varying values of synaptic connectivity parameters toward a meaningful mapping to Alzheimer disease-related EEG anomalies. However, such “synaptic parameter variation only” studies are highly constrained and do not make much sense when trying to understand generic brain-state conditions e.g., the sleep-awake cycle, or several other neurological and psychiatric disorders e.g., absence seizures, which rely heavily on various aspects of cellular dynamics in the thalamocortical circuitry. Rather, the emphasis of this work is on laying the ground-work for a more elaborate, and yet computationally efficient scheme, whereby large-scale computational models may be simulated to mimic brain rhythms, which can then be correlated to model parameters emulating cellular dynamics. The synaptic transmission kinetics and subsequent post-synaptic membrane parameters are some of the key constituents of brain signaling, and are affected significantly in various brain diseases. Clearly, the alpha-function based neural mass models are inadequate in dealing with research directions where model parameters can be mapped in a biologically plausible manner to synaptic attributes. In terms of computational efficiency, the time for simulating 20 trials with the model presented in this work takes 60 s; this may be contrasted with 600 s for simulating a similar model [the modified Alpha Rhythm model in Bhattacharya et al. (2011a)] based on alpha functions. This is a dramatic improvement in computational efficiency and highlight the plausibility of using the kinetic-model based neural mass modeling framework in simulating large-scale computational models toward mimicking real-time EEG signals. This in turn will provide a powerful tool for specifying cellular pathways that need be targeted for symptomatic alleviation of anomalous brain rhythms as well as to inform effective neuropharmacological research directions.

### Conflict of interest statement

The authors declare that the research was conducted in the absence of any commercial or financial relationships that could be construed as a potential conflict of interest.
